# Research Progress in Improving Photosynthetic Efficiency

**DOI:** 10.3390/ijms24119286

**Published:** 2023-05-26

**Authors:** Ruiqi Li, Ying He, Junyu Chen, Shaoyan Zheng, Chuxiong Zhuang

**Affiliations:** 1 Guangdong Laboratory for Lingnan Modern Agriculture, Guangzhou 510642, China; lrq0821@163.com (R.L.); 15876596509@163.com (Y.H.); mihal0220@163.com (J.C.); 2 State Key Laboratory for Conservation and Utilization of Subtropical Agro-Bioresources, College of Life Sciences, South China Agricultural University, Guangzhou 510642, China

**Keywords:** Calvin cycle, de novo synthesis, light reactions, non-photochemical quenching, photosynthetic efficiency, stomatal conductance

## Abstract

Photosynthesis is the largest mass- and energy-conversion process on Earth, and it is the material basis for almost all biological activities. The efficiency of converting absorbed light energy into energy substances during photosynthesis is very low compared to theoretical values. Based on the importance of photosynthesis, this article summarizes the latest progress in improving photosynthesis efficiency from various perspectives. The main way to improve photosynthetic efficiency is to optimize the light reactions, including increasing light absorption and conversion, accelerating the recovery of non-photochemical quenching, modifying enzymes in the Calvin cycle, introducing carbon concentration mechanisms into C_3_ plants, rebuilding the photorespiration pathway, de novo synthesis, and changing stomatal conductance. These developments indicate that there is significant room for improvement in photosynthesis, providing support for improving crop yields and mitigating changes in climate conditions.

## 1. Introduction

**Figure 1:**
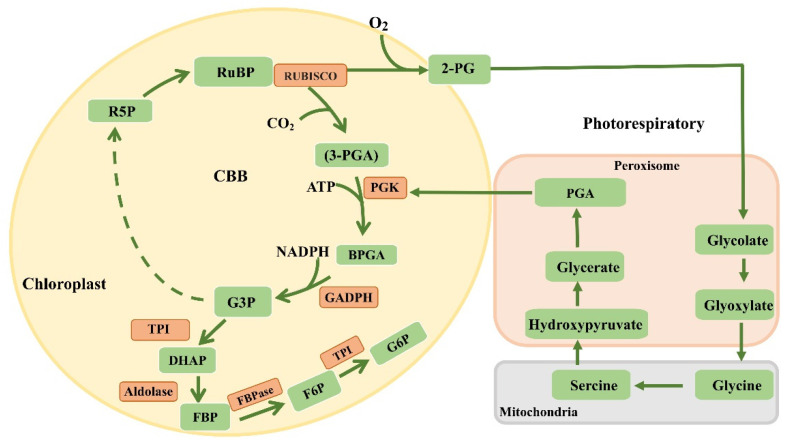
Carbon metabolism cycle diagram: the Calvin–Benson–Bassham (CBB) cycle and photorespiratory pathway. Rubisco, ribulose 1,5-bisphosphate carboxylase/oxygenase; 3-PGA, 3-phosphoglycerate; PGK, phosphoglycerate kinase; BPGA, glycerate 1,3-bisphosphate; GAPDH, glyceraldehyde 3-phosphate dehydrogenase; G3P, glyceraldehyde 3-phosphate; TPI, triose phosphate isomerase; DHAP, dihydroxyacetone phosphate; FBPase, fructose 1,6-bisphosphatase; F6-P, fructose 6-phosphate; GPI, glucose phosphate isomerase; Ru5P, ribulose 5-phosphate; 2PG, 2-phosphoglycerate; PGA, phosphoglycerate.

Photosynthesis refers to the process in which autotrophs convert light energy from the sun into chemical energy to convert inorganic carbon into complex organic carbon and release oxygen. Photosynthetic activity has enabled a large accumulation of organic matter and oxygen on Earth. Heterotrophic organisms use the organic matter and energy generated by photosynthesis to reproduce, grow, and evolve. The process of photosynthesis can be basically divided into two stages: light and dark reactions. The light-reactions stage includes the primary reaction, electron transport, and photophosphorylation; the dark-reactions stage is also called the carbon-assimilation reaction [1]. The process in which pigment molecules produce electrons by capturing solar energy is called photosynthetic electron transport (PET). PET includes linear electron transport (LET) and cyclic electron transport (CET). The light reactions of photosynthesis are the starting points of the whole photosynthesis process, providing adenosine triphosphate (ATP) and nicotinamide adenine dinucleotide phosphate (NADPH) as the energy sources for the subsequent dark reactions, and part of the ATP and NADPH generated enters the photorespiratory pathway (Figure 1). Carbon assimilation is the carbon-reaction stage of photosynthesis, which is essentially a process of inorganic carbon fixation and conversion to organic carbon. There are various ways to fix carbon, with most autotrophs utilizing the Calvin cycle.

In recent decades, new progress has been achieved in the study of the key mechanisms of photosynthesis, but the actual efficiency of photosynthesis in converting absorbed light energy into energy substances is very low compared to theoretical values. During light reactions, photosynthesis in autotrophs is limited by the light-capture range and electron-transport efficiency. During dark reactions, the efficiency of a series of enzyme activities is low, with stomatal and mesophyll limitations, and the efficiency of converting absorbed light energy into energy substances is very low compared with the theoretical value. Improving photosynthetic efficiency involves improving light capture and conversion capacities in the light-reaction stage, optimizing the electron-transport chain, increasing Rubisco carboxylase activity, and reducing photorespiration [2].

## 2. Light Reactions

**Figure 2:**
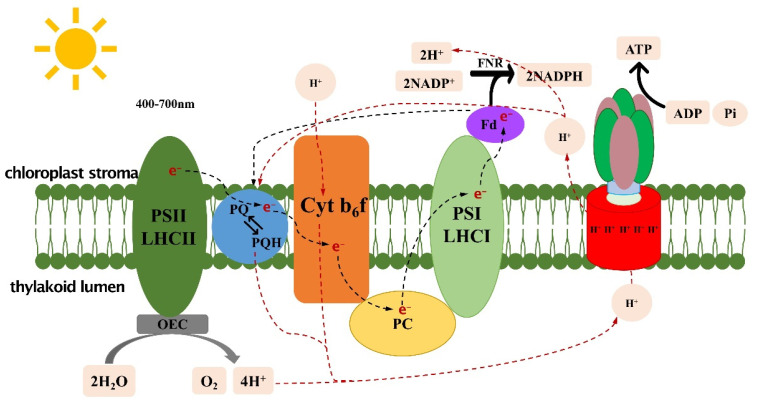
PSI-LHCI, photosystem I light-harvesting complex I; PSII-LHCII, photosystem II light-harvesting complex II; PQ, plastoquinone; PQH, semi-plastoquinone; PC, plastocyanin; Cytb6f, cytochrome *b*6*f*; Fd, ferredoxin; NAD(P)H dehydrogenase.

Light reactions occur at the chloroplast thylakoid membrane, and the reaction rate is related to the light intensity. Protein complexes on chloroplast thylakoid membranes can bind with a variety of photosynthetic pigment molecules, which absorb and transfer light energy and convert it to chemical energy (Figure 2). In higher plants, common pigment molecules include chlorophyll a, chlorophyll b, and carotenoids (β-carotene, lutein, violaxanthin, neoxanthin, etc.) [3]. These pigment molecules are excited by wavelengths of 400–700 nm in solar radiation [4] and enter the excited state from the ground state. The excited-state pigment molecules are electron donors and transfer high-energy electrons to nearby electron acceptors; the electrons obtained are coupled with the phosphate bond in the compound to convert electrical energy to chemical energy.

LET carries out classic “Z” transmission. First, the pigment P680 in the reaction center of photosystem II (PSII) is excited to produce a high-energy electron, which is transferred to the original electron acceptor magnesium-free chlorophyll. The electrons are further transferred to plastoquinone (PQ) and the cytochrome b6f complex (Cytb6f). The Cytb6f complex receives electrons and transfers H^+^ from the chloroplast stroma to the thylakoid lumen. Then, the electrons are transferred to plastocyanin (PC), which transfers the electrons to the reaction center pigment P700 of photosystem I (PSI). Finally, the electrons are transferred to ferredoxin (Fd), and NADP^+^ is converted to NADPH under the action of ferredoxin-NADP reductase (FNR) (Figure 2) [2]. In the process of LET, the H^+^ entering the thylakoid lumen from the chloroplast stroma and the H^+^ generated by photolysis of water establish the transmembrane proton potential. The chemical permeation hypothesis suggests that the chemical electromotive force of hydrogen ions inside and outside the membrane drives ATP synthesis under the action of ATP synthase [5]. CET refers to electron transfer from Fd back to PQ in plants lacking NADP^+^, which occurs around PSI. During this process, only ATP is synthesized, whereas NADPH is not [6] (Figure 2). Plants can change the proportion of ATP/NADPH in vivo to meet different metabolic needs through regulation between LET and CET. Research shows that after expression of CET-related genes is downregulated in plants, the photoprotection and electron transmission capacity are also significantly reduced [7]. Existing research has identified two CET pathways: the NDH (NADPH dehydrogenase)-dependent pathway [8] and the PGR5 pathway [7]. The electrons in the NDH-dependent pathway return from NDH to Fd and are then transferred to PQ and, finally, return to PSI through the Cytb6f complex and PC. After knocking out NDH in tobacco, PQ cannot be reduced, resulting in a decrease in chlorophyll fluorescence [8]. The PGR5 pathway was found in *Arabidopsis* mutant pgr5 and PGR5-like mutants [9,10]. The absence of the PGR5 complex in this mutant inhibits CET.

## 3. Increased Light-Capture Capability and Optimized Light Absorption and Conversion

Expanding the spectral range of plant photosynthesis and shortening the photosynthetic antenna complex are also important means to improve photosynthesis [11]. The effective spectral range of the light-harvesting pigment molecules in green plants is 400–700 nm, but the photosynthetic pigment molecules used by certain algae and other photosynthetic bacteria can capture and utilize near-infrared radiation with longer wavelengths (740–750 nm) [12]. Optimizing the antenna size of photosynthetic systems is one of the important directions to improve photosynthetic efficiency. Engineering and modification of pigment molecules in the light-harvesting system can increase solar-energy absorption to improve the photosynthetic rate, and with carbon assimilation efficiency also increase it with rising light-harvesting efficiency. The ATP and NADPH produced by electron transport in the photoreaction provide energy substances for the subsequent dark reactions. Under sufficient lighting conditions, the light energy that chlorophyll molecules can capture is much higher than the actual light energy utilized, and the excess absorbed light energy needs to be dissipated in other forms to avoid damage to the photosystems [13]. In leaves, this causes upper-surface cells to dissipate most of the light energy; however, the cells on the underside of the leaf lack sufficient absorption of light energy, resulting in low overall efficiency of light-energy absorption. For the whole plant, the upper leaves absorb most of the light energy [14]. Under current atmospheric conditions, shortening the antenna of light-harvesting pigment molecules can solve the problem of surface light saturation and lower-layer light deficiency. Theoretically, truncation of the chlorophyll antenna complexes of the photosystems might lead to a large increase in solar conversion efficiency [15]. Reducing chlorophyll content may also promote a more uniform distribution of light energy to increase the photosynthetic rate [16]. H. Kirst et al. shortened the antenna of photopigment molecules in cyanobacteria, improving the photosynthetic rate [17], and similar results were obtained in *Arabidopsis* [18]. These results suggest that shortening the antenna of photopigment molecules while maintaining the ability to adapt to fluctuating light is a potential strategy for improving photosynthesis. *Cao* encodes chlorophyll, which can affect the size of the antenna molecules of the photosystems [19], and the photosynthetic rate of *Cao* mutants obtained using RNAi technology increased more than two-fold compared with that of the wild type under strong light [20]. A shortened chlorophyll antenna also reduces non-photochemical quenching [21]. Inhibiting the *YGL1* gene involved in chlorophyll synthesis and inhibiting chlorophyll synthesis can reduce antenna size and optimize light distribution [22].

## 4. Accelerating Recovery of NPQ (Non-Photochemical Quenching)

Any excess light during photosynthesis can lead to photooxidative damage and reduce carbon assimilation. The process of excessive excitation energy in the form of thermal energy in the PSII antenna complex is called chlorophyll fluorescence non-photochemical quenching (NPQ). Reactive oxygen species disrupt photosynthetic devices [23], and NPQ protects PSII from photoinhibition by preventing reactive oxygen species formation [24]. Heat dissipation of excess energy is activated by the electron flow of CET through acidification of the cystoid cavity, which is the main component of NPQ [25]. The change in NPQ lags behind the fluctuation of absorbed light, which is more pronounced when exposed to excessive light energy for a long time, or repeatedly [26]. This lag causes the PSII antenna to slow from a quenching to nonquenching state when the plant is exposed to high to low light intensity, and the CO_2_ fixation rate is briefly suppressed by NPQ. Therefore, accelerating recovery of NPQ can significantly improve canopy photosynthesis, and controlling the photoprotection pathway may be a means to improve crop stress resistance and photosynthetic productivity [11]. LHCSR1 and LHCSR2 are the main proteins that induce NPQ. When either is mutated or both are knocked down simultaneously, *Chlamydomonas reinhardtii* photosynthetic efficiency under intense light increased significantly [27,28]. Overexpression of NPQ-related genes in tobacco, redesigning NPQ, successfully enhances photosynthesis during natural photoconversion, increasing plant biomass by 14–20% [29]. Results similar to those in *Arabidopsis* were also achieved in soybean. Faster relaxation of NPQ was obtained though redesign via overexpression of related genes in soybean, and most of the transgenic lines obtained showed an obvious increase in the rate of CO_2_ assimilation and linear electron transport under fluctuating light conditions [30]. Introduction of a soybean-specific xanthophyll cycle in *Arabidopsis* accelerated NPQ recovery, resulting in an increase in PSII efficiency and a concomitant increase in the photosynthetic rate [31]. These results indicate that modulating NPQ is a feasible strategy to adjust the photosynthetic rate.

## 5. Improving the Cytb6f Complex

In the CET pathway, H^+^ is transferred to the cystoid cavity through the Cytb6f complex. The protective effect of CET on PSI depends on ΔpH [32], which regulates electron transport by acidification of the thylakoid lumen [33,34]. Acidification of the thylakoid lumen reduces the electron transport of the Cytb6f complex, resulting in a decrease in the rate from PSII to PSI [32], thus realizing the regulation of the donor side of PSI. Overexpression of the Rieske FeS protein in *Arabidopsis* increased the core protein level of the Cytb6f complex, resulting in an increase in the electron-transport rate and biomass yield [35]. Overexpression of the Rieske FeS subunit in *Setaria viridis* and obtaining plants with high expression of Cytb6f in mesophyll and bundle sheath cells increased the conversion efficiency of the light system and the thylakoid proton kinetic energy, improving the CO_2_ assimilation efficiency under saturated CO_2_ and strong light [36].

## 6. Dark Reactions

Carbon assimilation is the carbon-reaction stage of photosynthesis, which is essentially a process of inorganic carbon fixation and conversion to organic carbon. There are various ways to achieve carbon fixation, and most autotrophs use the Calvin cycle. Other pathways include the ribose–monophosphate pathway [37] and the 3-hydroxy-propionate pathway [38]. The Calvin cycle, also known as the C_3_ pathway, occurs in the chloroplast stroma. The process can be divided into CO_2_ fixation, reduction of carboxylation products, and regeneration of ribulose 1,5-bisphosphonate (RuBP). First, CO_2_ combines with RuBP under catalysis of ribulose 1,5-diphosphate carboxylase/oxygenase (Rubisco) carboxylase to form a highly unstable six-carbon compound, which randomly splits to form two molecules of 3-phosphoglycerate (PGA). Then, 3PGA receives a phosphoric acid molecule from ATP in the reaction under catalysis of phosphoglycerate kinase (PGK) to generate 1,3-diphosphoglyceride (BPGK); glyceraldehyde 3-phosphate dehydrogenase (GADPH) generates glyceraldehyde 3-phosphate (G3P) from 1,3-diphosphoglyceride and NADPH. C_3_ plants generate two molecules of G3P for every one molecule of CO_2_ fixed. However, only for every six molecules of G3P generated can one molecule of G3P enter subsequent carbon fixation, and the remaining five molecules of G3P are used for RuBP regeneration. G3P is isomerized to dihydroxyacetone phosphate (DHAP) by triose phosphate isomerase; DHAP generates fructose 1,6-diphosphate (FBP) under aldolase condensation; FBP loses one Pi molecule under catalysis of fructose diphosphatase to generate fructose 6-P (F6P); F6P is allosterically transformed to glucose 6-phosphate (G6P) under catalysis of glucose isomerase; G6P is used to form starch through a series of transformations. The Calvin cycle generates a molecule of glucose via fixation of six molecules of CO_2_, converting the chemical energy of ATP and NADPH generated in the light reactions to stable organic matter and using the energy supply of subsequent physiological and biochemical processes (Figure 1).

## 7. Modification of Rubisco

Rubisco is the most critical rate-limiting enzyme for carbon fixation in photosynthesis, playing a decisive role in the carbon-reaction rate. Rubisco is a bifunctional enzyme that not only catalyzes carbon fixation in carboxylation reactions but also produces toxic substances through oxidation reactions that need to be metabolized through photorespiration (Figure 1) [39]. In plants, Rubisco comprises the highest soluble protein content, accounting for three-quarters of all soluble proteins in C_3_ plants. However, its catalytic efficiency is very low, and its substrate specificity is also poor [40], which is why plants need a high Rubisco content to ensure high-speed photosynthesis. Theoretically, improving the very low catalytic activity of Rubisco can greatly improve the photosynthetic efficiency of crops [41]; hence, improving Rubisco in crops to achieve better performance is a focus of researchers. Indeed, heterologous expression of Rubisco is an important means to improve photosynthesis. Expressing the sorghum Rubisco small subunit in rice resulted in stronger catalytic activity [42]. When the Rubisco subunit of sorghum was completely replaced by the Rubisco subunit of rice, the transgenic plants obtained exhibited the characteristics of C_4_ plants, and the mechanical parameters related to Rubisco enzyme activity significantly increased [43]. Rubisco from *Synechococcus elongatus PCC7942* introduced into tobacco can completely assemble, possesses functional activity and photosynthetic ability, and supports autotrophic growth [44]. Overexpression of Rubisco in rice led to higher photosynthetic capacity under sufficient nitrogen conditions [45]. Scientists have determined the effect of mutations on Rubisco assembly and activity through point mutation of the Rubisco large subunit in tobacco, providing more possibilities for altering Rubisco function to improve photosynthesis [46].

## 8. Optimization of Enzymes in the Calvin Cycle

The essence of the Calvin cycle is a series of enzyme catalysis. Therefore, in addition to improving the activity of Rubisco carboxylase, other enzymes in the Calvin cycle are important for improving photosynthetic efficiency. Carbamylation of Rubisco activase (RCA), that is, addition of carbon dioxide at the active site of lysine, is a prerequisite for activation of Rubisco [47], but premature binding of RuBP or other sugar phosphates may hinder its activity [48,49]. As the thermal instability of RCA can inhibit carbon fixation under thermal stress conditions [50], modification of RCA is also an important goal for enhancing photosynthesis. For example, increasing the thermal stability of *Arabidopsis* RCA can increase photosynthesis, growth rate, and biomass under moderate heat stress [51]. The more heat-resistant tobacco RCA not only has a higher photosynthetic rate under high-temperature conditions but also recovers better under normal-temperature conditions, with increased biomass and seed yield [52]. Similarly, overexpression of maize RCA in rice results in a higher activation state of Rubisco under weak light and a faster photosynthetic response when light intensity increases [53]. Transgenic plants overexpressing Rubisco and RCA in rice also have higher CO_2_ assimilation rates [54].

Sedoheptulose-1,7-bisphosphatase (SBPase) is an important enzyme in the Calvin cycle. It is predicted that as the concentration of carbon dioxide in the atmosphere increases, an increase in SBPase content will be beneficial for plants [55], and recent research has shown the accuracy of the prediction. For example, upregulation of the content of this enzyme in tobacco significantly improves tobacco photosynthesis and yield, and this increase in photosynthesis is more pronounced under high concentrations of carbon dioxide [56]. The photosynthetic efficiency and biomass of wheat also increases significantly when SBPase is overexpressed [57].

## 9. Introduction of Carbon Concentration Mechanisms into C_3_ Plants

According to the process of CO_2_ fixation with Rubisco, photosynthesis is mainly divided into three metabolic pathways: C_3_, C_4_, and CAM. Among them, the carbon concentration mechanisms (CCMs) evolving from C_4_ and CAM metabolism are conducive to increasing Rubisco carboxylase activity while limiting its photorespiration rate [58]. C_4_ plants are more efficient in utilizing light, nitrogen, and water than C_3_ plants [1]. CAM can increase the CO_2_ concentration near that of Rubisco while also reducing water evaporation and increasing water-use efficiency. Therefore, introducing C_4_ or CAM photosynthetic metabolic pathways into C_3_ plants is one of the methods to enhance photosynthesis. Mesophyll cells and vascular bundle sheath cells are two types of cells that carry out photosynthesis in C_4_ plants. Multiple layers of mesophyll cells surround concentrically arranged vascular bundle sheath cells to form a floral structure (Kranz anatomy). During carbon metabolism of C_4_ plant photosynthesis, the carboxylic acid transformation of phosphoenolpyruvate (PEP) in mesophyll cells and vascular bundle cells and continuous regeneration through transportation generates inorganic carbon with a 10-times higher concentration than that of Rubisco [59], forming CCMs; thus, the plant has higher photosynthetic efficiency. Overexpression of maize-related genes in rice leads to an increase in accumulation of photosynthetic enzymes, which means that C_3_ plants can be transformed to C_4_ plants [60]. Overexpression of the maize transcription factor *GLK* in rice improves photosynthetic efficiency [61]. Scientists have also confirmed that constructing C_3_-C_4_ intermediate metabolism or introducing a carboxyl matrix can improve productivity [62]. Lin et al. used tobacco as a model system to transfer two molecular chaperones from cyanobacterium *Synechococcus elongatus PCC794*2 (Se7942) into C_3_ plant chloroplasts. Tobacco can grow under high concentrations of CO_2_, which is an important step in introducing CCMs into vascular plant chloroplasts [63]. Long et al. successfully replaced the large and small subunit genes of Rubisco from *Cyanobium* with the endogenous large subunit genes of Rubisco in the chloroplast of tobacco, achieving autotrophic growth under conditions of high carbon dioxide [64] and providing a possibility for CCM engineering of C_3_ crop chloroplasts and improving photosynthesis. Transfer of the Rubisco large subunit of *β-cyanobacterium* into the Rubisco complex with the carboxyl chaperone CcmM35 in tobacco chloroplasts further demonstrates the possibility of assembling these complex CCMs in plants [65]. Rubisco derived from *Halothiobacillus neapolitanus* was expressed in tobacco chloroplasts, leading to the same growth rate as wild-type plants at a concentration of 1% CO_2_, indicating that CCMs can be applied to higher-plant Rubisco, which is an important step toward improving crop photosynthesis [66].

## 10. Reconstructing the Photorespiratory Pathway

Photorespiration refers to the process in which RuBP binds O_2_ under catalysis of Rubisco to generate 2-phosphoglycerate (2-PG) and then generate PGA (Figure 1). As photorespiration causes a large amount of carbon loss (Figure 1), reforming the photorespiratory pathway is also an important method to improve photosynthesis [40,67]. Kebeish et al. [68] were the first to introduce three enzymes involved in glycolic acid metabolism in *Escherichia coli* into the chloroplasts of *Arabidopsis*, successfully shunting glycolic acid in the chloroplasts and releasing CO_2_, but this approach can only improve biomass under specific conditions. A photorespiratory pathway that does not release CO_2_ and enhances carbon fixation was established in vitro by combining natural and artificially designed glycolate reductase [69], but this design is limited to in vitro simulation and has not been verified *in planta*. In tobacco, RNAi has been used to downregulate expression of glycolate transporters in chloroplasts, thereby limiting metabolites through its own pathway, reducing transport expression of glycolate and glyceric acid, and increasing the biomass of plants under field conditions by 40% [70]. Shen et al. (2019) designed a new photorespiratory pathway in rice by introducing glycolate oxidase, oxalate oxidase, and catalase into chloroplasts to enable glycolate to directly metabolize and release CO_2_ in the chloroplasts, that is, the photorespiratory GOC pathway. This approach significantly improved the photosynthetic efficiency and biomass of the rice plants. These results provide reliable evidence that reconstructing the photorespiratory pathway can improve the photosynthetic efficiency of rice [71].

## 11. Redomestication/De Novo Domestication

Genetic diversity has decreased over thousands of years of plant evolution. *De novo* domestication refers to the cultivation of new species from wild species [72]. Obtaining specific genes from wild plants and transferring them to cultivated plants to restore lost traits, resulting in new varieties, is called redomestication [73,74,75]. After determining the main genes involved in the regulation of photosynthesis, plants with high photosynthetic efficiency can be designed rapidly by means of de novo acclimation and genome-editing technology [76]. Transcription factors are an important target in redomestication, such as overexpression of the maize MADS-box transcription factor gene *zmm28*, which ultimately leads to an increase in growth, photosynthetic capacity, and nitrogen utilization [77]. With advances in genetic engineering, genome editing and synthetic biology are increasingly being widely used in various plants [78]. Scientists have determined the molecular genetic mechanism and, ultimately, a target gene by analyzing postrepresentative types of wild type and domesticated plant hybrids. For example, 14 genes directly related to the photosynthetic rate were identified by crossing 76 offspring of wild-type tomato and cultivated tomato *cv M82* [79], and identification of these genes may serve as a direct goal for improving photosynthesis.

## 12. Changes in Stomatal and Mesophyll Conductance

In C_3_ plants, stomatal conductance (gs) and mesophyll conductance (gm) are important influencing factors for photosynthesis [80]. Plants absorb CO_2_ for photosynthesis, and gs and gm are the factors determining CO_2_ diffusion efficiency [81]. Gs and gm control the photosynthetic rate by affecting the CO_2_ concentration at carboxylation sites [82,83]. Especially under stress conditions, stomatal limitations have a higher impact on photosynthesis than biochemical limitations, such as Rubisco limitations [84]. Increasing stomatal conductance is an important means to improve photosynthetic efficiency [85]. For example, stomata are the main limiting factor for photosynthesis during the adaptation process from darkness to bright light in tobacco and *Arabidopsis* [86]. Stomata control absorption and transpiration of carbon dioxide at the same time, and scientists can achieve a balance between the two by changing the size and shape of the stoma and the density and distribution of mesophyll cells, thus improving photosynthesis. In fact, changing the size, shape, density, and distribution of stomata are some of the important means to improve photosynthesis [83,85]. The gene *ERECTA*, which regulates transpiration efficiency, can change the density, distribution, and intercellular contact to increase the rate of CO_2_ absorption [87]. *NAL1* in rice thickens leaves by increasing the number of mesophyll cells, resulting in a significant increase in photosynthesis [88]. Increasing the density of mesophyll cells in *Arabidopsis* also significantly improves the photosynthetic efficiency of leaves [89]. Changing the thickness of the cell wall to affect stomatal conductance and mesophyll conductance will also be important research directions to improve photosynthetic efficiency in the future [80]. By knocking out or overexpressing genes related to regulation of stomatal development in *Arabidopsis*, increasing stomatal density significantly increases stomatal conductance and leaf photosynthetic rate [90]. Moreover, the open and closed states of stomata are important factors in improving photosynthetic efficiency. In mutants obtained by knocking out *SLAC1*, photosynthetic efficiency and stomata opening in rice were significantly higher than that of wild-type [91].

## 13. Conclusions

**Figure 3:**
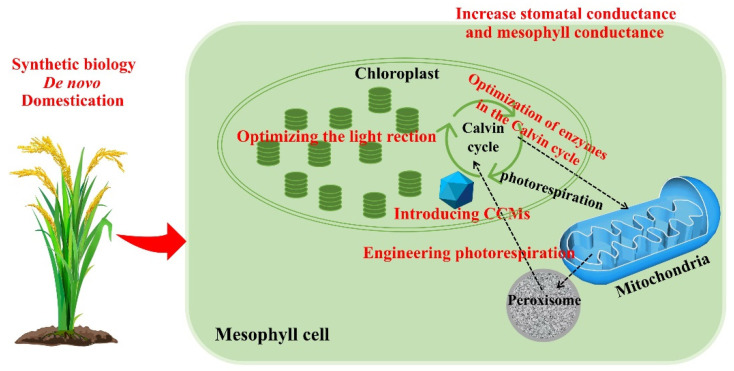
Ways to improve photosynthetic efficiency: optimizing light reactions; optimizing enzymes in the Calvin cycle; introducing CCMs; engineering photorespiration; synthetic biology; de novo domestication; increasing stomatal conductance and mesophyll conductance.

Photosynthesis is one of the most important physiological and biochemical processes on Earth, but because of the limitations of the photosynthetic autotroph system, the overall efficiency of the process is not high. Based on these restrictive factors of photosynthetic efficiency, scientists are attempting to combine genomics, transcriptomics, proteomics, metabolomics, and other methods with genome editing to improve the photosynthetic efficiency of autotrophic plants (Figure 3). The challenges involved are enormous and numerous, but the progress achieved by scientists is encouraging. In-depth study of the molecular mechanism of photosynthesis will help in understanding photosynthesis more deeply and improving the photosynthetic efficiency of plants, especially the photosynthetic efficiency of food crops, which will bring infinite possibilities for improving the yield of food crops. Photosynthetic regulation is a complex process that must be comprehensively considered to reduce negative impacts on plants, organisms, and humans.

## Data Availability

Not applicable.

## Abbreviations

ATP	adenosine triphosphate
BPGA	glycerate 1,3-bisphosphate
CBB	Calvin–Benson–Bassham
CCMs	carbon concentration mechanisms
CET	cyclic electron transport
Cytb6f	cytochrome b6f
DHAP	dihydroxyacetone phosphate
FBPase	fructose 1,6-bisphosphatase
Fd,	ferredoxin
F6-P	fructose 6-phosphate
GAPDH	glyceraldehyde 3-phosphate dehydrogenase
gm	and mesophyll conductance
GPI	glucose phosphate isomerase
gs	stomatal conductance
G3P	glyceraldehyde 3-phosphate
LET	linear electron transport
NADPH	nicotinamide adenine dinucleotide phosphate
NPQ	Non-Photochemical Quenching
PC	plastocyanin
PET	photosynthetic electron transport
PEP	phosphoenolpyruvate
PGK	phosphoglycerate kinase
PGA	phosphoglycerate
PQ	plastoquinone
PQH	semi-plastoquinone
PSI	photosystem I
PSI-LHCI	photosystem I light-harvesting complex I
PSII	photosystem II
PSII-LHCII	photosystem II light-harvesting complex II
RuBP	ribulose 1,5-bisphosphonate
Rubisco	ribulose 1,5-bisphosphate carboxylase/oxygenase
Ru5P	ribulose 5-phosphate
SBPase	Sedoheptulose-1,7-bisphosphatase
TPI	triose phosphate isomerase
2PG	2-phosphoglycerate
3-PGA	3-phosphoglycerate
